# PAK1 Kinase Promotes Cell Motility and Invasiveness through CRK-II Serine Phosphorylation in Non-Small Cell Lung Cancer Cells

**DOI:** 10.1371/journal.pone.0042012

**Published:** 2012-07-27

**Authors:** Matthew Rettig, Kenny Trinidad, G. Pezeshkpour, Patrick Frost, Sherven Sharma, Farhad Moatamed, Fuyuhiko Tamanoi, Fariborz Mortazavi

**Affiliations:** 1 Division of Hematology/Oncology, West Los Angeles VA, Los Angeles, California, United States of America; 2 Department of Medicine, University of California Los Angeles, Los Angeles, California, United States of America; 3 Jonsson Comprehensive Cancer Center, Los Angeles, California, United States of America; 4 Department of Pathology, West Los Angeles VA, Los Angeles, California, United States of America; 5 Division of Molecular Medicine, West Los Angeles VA, Los Angeles, California, United States of America; 6 Department of Microbiology, Immunology and Molecular Genetics, University of California Los Angeles, Los Angeles, California, United States of America; Aix-Marseille University, France

## Abstract

The role of c-Crk (CRK) in promoting metastasis is well described however the role of CRK phosphorylation and the corresponding signaling events are not well explained. We have observed CRK-II serine 41 phosphorylation is inversely correlated with p120-catenin and E-cadherin expressions in non-small cell lung cancer (NSCLC) cells. Therefore, we investigated the role of CRK-II serine 41 phosphorylation in the down-regulation of p120-catenin, cell motility and cell invasiveness in NSCLC cells. For this purpose, we expressed phosphomimetic and phosphodeficient CRK-II serine 41 mutants in NSCLC cells. NSCLC cells expressing phosphomimetic CRK-II seine 41 mutant showed lower p120-catenin level while CRK-II seine 41 phosphodeficient mutant expression resulted in higher p120-catenin. In addition, A549 cells expressing CRK-II serine 41 phosphomimetic mutant demonstrated more aggressive behavior in wound healing and invasion assays and, on the contrary, expression of phosphodeficient CRK-II serine 41 mutant in A549 cells resulted in reduced cell motility and invasiveness. We also provide evidence that PAK1 mediates CRK-II serine 41 phosphorylation. RNAi mediated silencing of PAK1 increased p120-catenin level in A549 and H157 cells. Furthermore, PAK1 silencing decreased cell motility and invasiveness in A549 cells. These effects were abrogated in A549 cells expressing phosphomimetic CRK-II serine 41. In summary, these data provide evidence for the role of PAK1 in the promotion of cell motility, cell invasiveness and the down regulation of p120-catenin through CRK serine 41 phosphorylation in NSCLC cells.

## Introduction

Development of metastatic lesions in most solid tumors result in an incurable condition by today’s treatment modalities. Therefore, understanding the events that promote tumor invasion and metastasis will help us prevent the spread of malignant tumors especially in case of early stage and perhaps oligometastatic disease. As a member of adherens junction, p120-catenin (p120ctn) plays an important role in cell-cell adhesions and loss of p120-catenin expression results in destabilization of the cadherin-catenin complex thereby promoting tumor invasion and metastasis [1], [2], [3], [4], . Considering downregulation of p120-catenin in non-small cell lung cancer (NSCLC) is transcriptionally mediated [11], an investigation in the upstream signaling events that could result in transcriptional repression of *p120-catenin (CTNND1),* lead us to adaptor protein CRK and its role in the repression of p120-catenin [12].

c-Crk (CRK) belongs to a family of widely expressed adaptor proteins that are involved in signal transduction from a variety of receptors and oncoproteins (e.g., Bcr-Abl; Tel-Abl, Erythropoietin receptor, EGFR, GM-CSF, Insulin receptor substrate, PDGF and VEGFR [13], [14]). CRK, interacts with several downstream effectors including SOS1, DOCK1 JNK1 and SP1. Our data show that CRK negatively regulates *p120-catenin (CTNND1)* transcription in NSCLC cells through interaction with transcription factor SP1 [12]. The central position of CRK in signaling cascades makes it likely that CRK affects several downstream targets, other than the *p120-catenin (CTNND1)* promoter, thereby promoting tumor progression, invasion and metastasis.

In addition to CRK overexpression as a proto-oncogene and its role in cell transformation, CRK phosphorylation is also apt to contribute to its biochemical activity. As an adaptor protein, CRK does not contain a catalytic domain however both tyrosine and serine kinase activities have been associated with CRK [15]. Proteins with phosphorylated tyrosine, serine or threonine residues were detectable in the immunoprecipitate of CRK in avian sarcoma virus (CT10) infected cells [15]. In this study, the product of CT10 virus (p47***^gag-crk^***) itself was also highly phosphorylated mainly on serine and about five percent on tyrosine residues. Several intracellular signaling pathways contribute to CRK phosphorylation. For example, c-Abl phosphorylates Crk on tyrosine 221 causing disassociation of Crk from the Crk-associated substrate (CAS) thereby inhibiting cell migration and promoting apoptosis in normal and malignant cells [16], [17]. Abl has a critical function in the formation and maintenance of adherens junctions as was shown in mouse embryonic fibroblasts [18], [19]. This effect of Abl on adherens junctions seems to be mediated via the Crk and Rac1 pathway that regulate cadherin-catenin adhesion complex. These findings are pointing to the fact that CRK phosphorylation plays an important role in cell motility and metastasis promotion.

With regards to CRK serine phosphorylation, two serine/threonine kinases, [i.e., hematopoietic progenitor kinase 1 (HPK1; MAP4K1) and kinase homologous to SPS1/STE20 (KHS;MAP4K5)], members of PAK serine/threonine kinase super family, are known to mediate phosphorylation of CRK family members and possibly participate in CRK mediated JNK activation [20], [21]. P21-activated kinases (PAKs) are well-known regulators of cytoskeletal remodeling and cell motility and it seems that PAK kinases play a role in metastatic promotion in malignant tumors [22]. For example, endogenous PAK is constitutively activated in certain breast cancer cell lines [23] and ectopic expression of constitutively activated PAK1 in nonmetastatic MCF-7 breast carcinoma cells increased cell motility [24]. In addition, overexpression of PAK1 was recently reported in NSCLC, mostly in squamous cell histology [25]. The above mentioned strongly suggest a role for CRK serine phosphorylation in metastatic promotion of malignant tumors. Biochemical pathways that regulate CRK serine phosphorylation and the role of CRK serine phosphorylation in metastasis promotion are not well studied to date. Here we provide evidence that PAK1 kinase mediates CRK-II serine 41 phosphorylation thereby promoting cell motility and invasiveness in NSCLC cells.

## Results

### CRK-II Serine 41 Phosphorylation is Inversely Correlated with *p120-catenin* Expression

**Figure 1:**
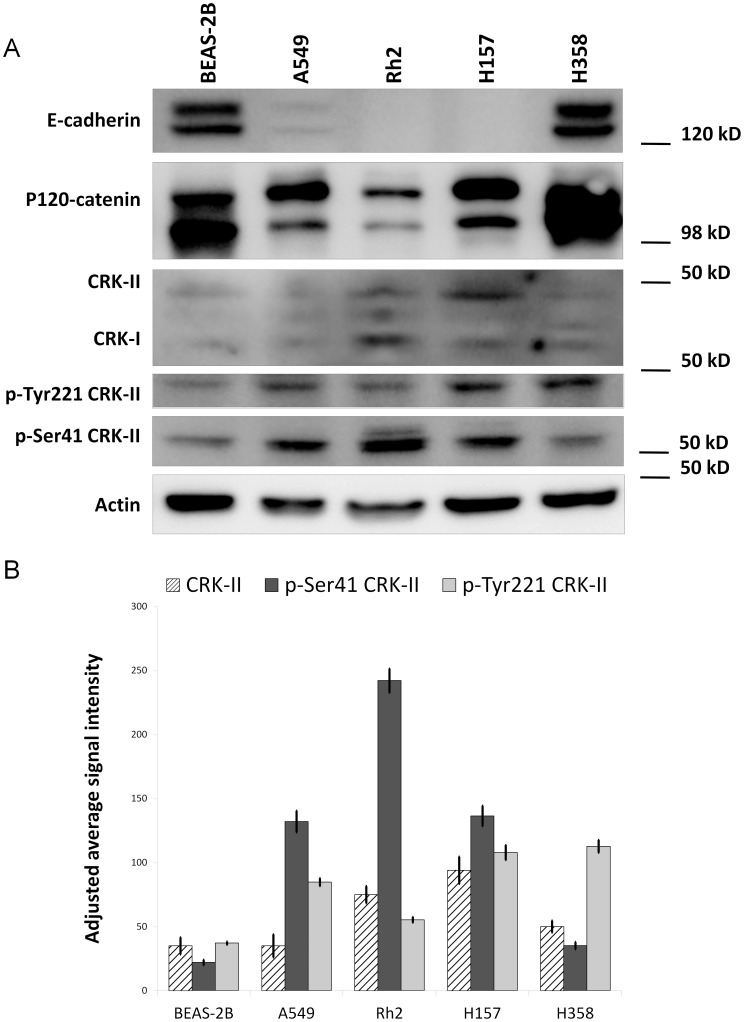
A- Western blots showing the expression pattern of p120-catenin, E-cadherin, CRK-I, CRK-II, phospho-serine 41 CRK-II and phospho-tyrosine 221 CRK-II in a panel of NSCLC cells and BEAS-2B cells. B- Quantification of CRK-II, phospho-tyrosine 221 CRK-II and phospho-serine 41 CRK-II signal intensity among NSCLC cell lines and BEAS-2B cells.

We recently reported that CRK mediates transcriptional repression of *p120-catenin (CTNND1)* in NSCLC cells. CRK can be phosphorylated in both tyrosine and serine residues, which then influences its interaction with downstream targets [15], [16], [26], [27]. Therefore, we sought to further investigate whether CRK phosphorylation status, as a surrogate of activated upstream signals, is possibly playing a role in CRK mediated *p120-catenin (CTNND1)* transcriptional repression. A close correlation of either serine or tyrosine CRK phosphorylation with p120-catenin expression would indicate the presence of an upstream serine/threonine or a tyrosine kinase that would mediate CRK phosphorylation thereby p120-catenin downregulation. For this purpose, we examined the phosphorylated CRK-II level both on tyrosine 221 and serine 41 and correlated that with p120-catenin levels in a panel of NSCLC and BEAS-2B cells (Figure 1). In case of tyrosine 221, phosphorylation of this residue by c-Abl is reported to result in cell migration inhibition. We observed an inverse correlation between phospho-serine 41 CRK-II with that of p120-catenin and E-cadherin protein levels by western blotting. Interestingly, phosphorylation of CRK-II on Y221 did not correlate with p120-catenin expression. These findings suggest that signaling events that engage serine/threonine kinases are involved in *p120-catenin (CTNND1)* transcriptional downregulation through serine phosphorylation of the CRK adaptor protein.

### CRK-II Serine 41 Phosphorylation Regulates *p120-catenin* Expression

**Figure 2:**
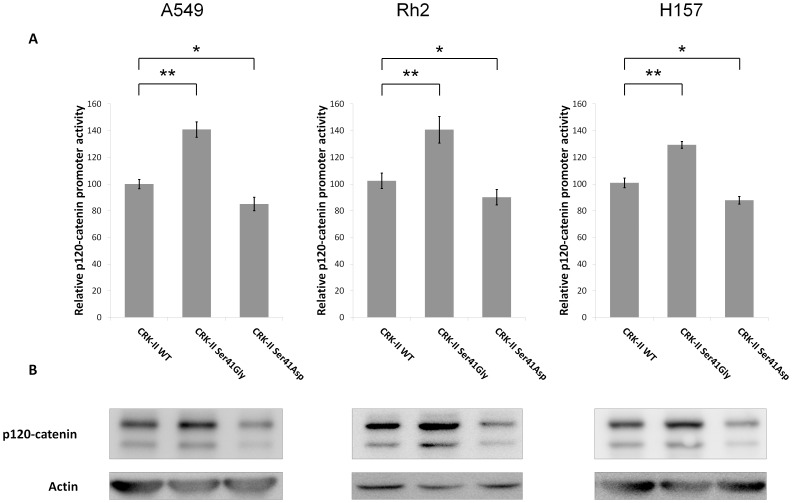
A- Relative *p120-catenin (CTNND1)* promoter activity in A549, Rh2 and H157 cell lines following transient transfection of CRK-II, CRK-II (Ser41Gly) or CRK-II (Ser41Asp) mutants. (2 tailed student’s t-test: * P<0.05; ** P<0.01; error bars represent ± standard deviation). B- Western blots showing changes in p120-catenin protein level in the above mentioned cell lines following transient transfection of CRK-II and CRK-II mutants.

In order to further assess the role of CRK serine 41 phosphorylation in the transcriptional regulation of *p120-catenin (CTNND1)*, we decided to examine whether CRK-II serine 41 manipulations might have any effect on the p120-catenin expression level. Therefore, we proceeded to prepare CRK-II serine 41 phosphodeficient and phosphomimetic mutants. A site directed mutagenesis reaction was utilized to replace CRK-II serine 41 with either Glycine [Ser41Gly (phosphodeficient)] or Aspartic acid [Ser41Asp (phosphomimetic)]. A CRK-II construct fused with a Myc-tag was used as the backbone of the site directed mutagenesis reactions therefore, all resulting mutants contained a Myc tag as well. All constructs were checked by sequencing. Subsequently, A549, Rh2 and H157 cells were transiently transfected with the resulting CRK-II mutants and we measured the *p120-catenin (CTNND1)* promoter activity as well as p120-catenin protein level by western blotting in the transfected cells. The expression levels of CRK-II mutants and the endogenous CRK-II are presented in (Figure S1). Compared to cells that expressed wild type CRK-II, a significant increase in *p120-catenin (CTNND1)* promoter activity was observed in cells expressing phosphodeficient CRK-II. On the other hand, a decrease in *p120-catenin (CTNND1)* promoter activity was observed in cells expressing phosphomimetic CRK-II serine 41 mutant (Figure 2). p120-catenin protein level also changed following expression of CRK-II mutants concordant to the observed changes in *p120-catenin* promoter activity alterations in all cell lines. These findings further emphasize in the role of CRK serine 41 phosphorylation in transcriptional regulation of *p120-catenin (CTNND1)*.

### CRK-II Serine 41 Phosphorylation is Involved in NSCLC Cell Motility and Cell Invasion

**Figure 3:**
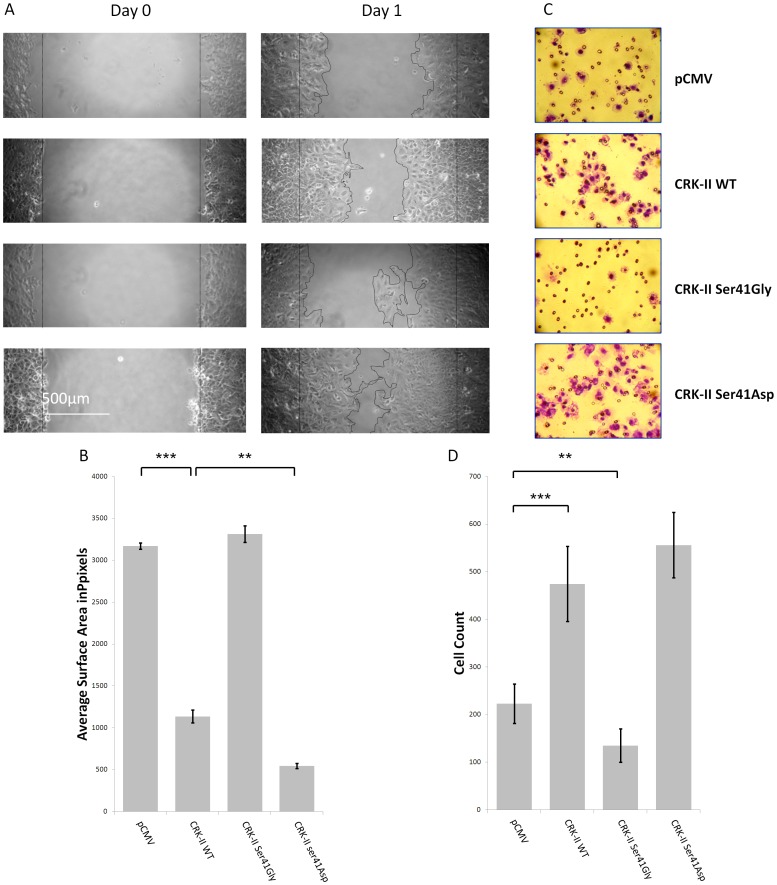
A-Wound healing assays in A549 cells stably expressing pCMV vector, wild type CRK-II (WT), CRK-II (Ser41Gly) or CRK-II (Ser41Asp) mutants. Endogenous CRK is silenced in all conditions by siRNA. B- Measurement of wound surface area 24 hours after establishment of the wound among the above mentioned groups. The average is calculated following measurement of three separate experiments. (2 tailed student’s t-test: ** P<0.01; *** P<0.001; error bars represent ± standard deviation).C- Invasion assays following incubation of stably transfected A549 cells expressing either pCMV vector; wild type CRK-II (WT); CRK-II (Ser41Gly) or CRK-II (Ser41Asp) mutants. 1×10^5^ cells were incubated over night as described in the Methods section and stained at 24 hours after incubation. D- Quantitative measurement of the invaded cells. Five separate sections of the invaded cells were counted. (2 tailed student’s t-test: ** P<0.01; *** P<0.001; error bars represent ± standard deviation).

Even though we observed CRK-II serine 41 phosphorylation is engaged in *p120-catenin (CTNND1)* transcriptional regulation, it is not clear whether phosphorylation of CRK-II on serine 41 has any other significant biological relevance. Since expression of CRK has been associated with more aggressive NSCLC tumors [28], we decided to examine the motile and invasive properties of A549 cells following manipulation of CRK-II serine 41 phosphorylation. Therefore, we proceeded to prepared A549 cells that stably express our CRK-II serine 41 mutants. A549 cells were chosen for this experiment since they express relatively low level of endogenous CRK-II. As described in the Material and Methods section, A549 cells were transfected with CRK-II serine 41 phosphodeficient and phosphomimetic mutants and were selected in the presence of G418. The pooled selected cells were used for the subsequent wound healing and cell invasion assays. The expression level of the mutant proteins were checked in a western blot assay (Figure S2). Next, we used the resulting A549 cells that stably expressed CRK-II serine 41 phosphodeficient and phosphomimetic mutants in wound healing and Matrigel cell invasion assays as explained in the Material and Methods section (Figure 3). As expected, compare to A549 cells expressing empty vector, wild type CRK-II expressing cells showed a more aggressive behavior. Interestingly, A549 cells expressing CRK-II phosphodeficient (Ser41Gly) mutant had a less motile property, almost similar to empty vector group and cells expressing phosphomimetic (Ser41Asp) mutant had the most aggressive behavior in the wound healing assays. We silenced the endogenous CRK by siRNA in all conditions in order to reduce the interference of the endogenous CRK with CRK-II mutant proteins (Figure S2). The siRNA that was used for silencing the endogenous CRK was directed against a sequence outside CRK’s open reading frame therefore this siRNA had no effect on the expression of CRK mutants by plasmid constructs. A wider view of this wound healing assay and the Matrigel membranes are presented in (Figures S3,S4). Similar to the wound healing assays, A549 cells expressing CRK-II serine 41 phosphodeficient mutant had less invasiveness compared to cells expressing empty vector and cells expressing CRK-II serine 41 phosphomimetic mutant had the most invasive property at 24 hours. Even though the endogenous CRK-II was incompletely silenced among the groups, the ratio of CRK-II mutants to the endogenous CRK-II was increased following silencing the endogenous CRK. It is worth mentioning these biological effects were not seen if the endogenous CRK was not silenced.

In order to examine whether changes in cell proliferation are contributing to the observed biological behavior of the cell lines with CRK-II mutants, cell proliferation rate of the resulting cell lines were measured (Figure S5). Following plating equal number of A549 cells transfected with CRK-II and CRK-II mutants, cells were counted at 24 hour intervals. No significant difference in cell numbers were noted among the groups. These observations corroborate the involvement of CRK-II serine 41 phosphorylation in the motility and invasiveness of NSCLC cells.

### PAK1 kinase Mediates CRK-II Serine 41 Phosphorylation

**Figure 4:**
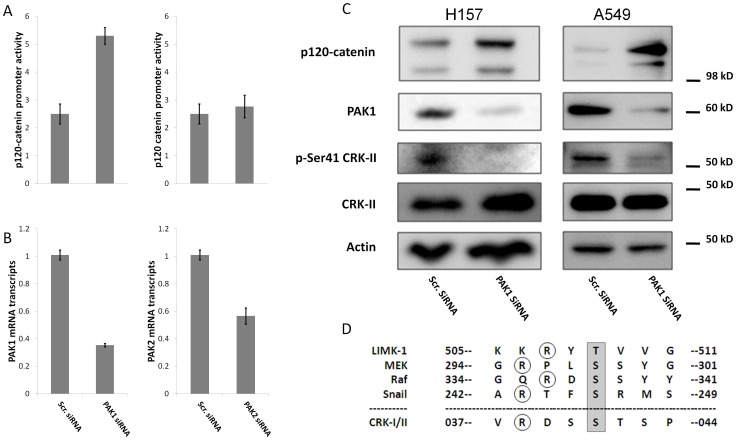
A-Relative *p120-catenin (CTNND1)* promoter activity in A549 cells following siRNA mediated silencing of PAK1 and PAK2. B- Quantitative real time PCR measurement of PAK1 and PAK2 mRNA in order to determine the silencing efficiency of siRNA. C-Western blots showing phospho-serine 41 CRK-II and p120-catenin expression following siRNA mediated PAK1 silencing in A549 and H157 cells. D- Pak1 phosphorylation consensus sequence in several Pak1 substrates. Residues that are phosphorylated by Pak1 are highlighted in gray. Circled are the upstream arginine residues that are important for Pak1 target phosphorylation.

Considering the role of CRK-II serine phosphorylation on the *p120-catenin (CTNND1)* transcriptional repression and also promotion of motility and invasiveness in NSCLC cells, we sought to identify the upstream kinase(s) that might mediate CRK-II serine 41 phosphorylation. CRK serine phosphorylation has been reported by several members of the PAK serine/threonine family of kinases [20], [21]. For example, Hematopoietic Progenitor Kinase 1 (HPK1; MAP4K1) and Kinase Homologous to SPS1/STE20 (KHS; MAP4K5) are reported to phosphorylate CRK family members on serine residues. Therefore, we decided to examine whether CRK-II serine 41 phosphorylation might be mediated by PAK family of kinases. Towards this end, we chose to silence PAK1 by siRNA in H157 and A549 NSCLC cells and examine CRK-II serine 41 phosphorylation by western blotting. Compared to cells treated with non-silencing siRNA, PAK1 silencing lead to dramatically reduced phospho-serine 41 CRK-II in both H157 and A549 cells (Figure 4C). Furthermore the amino acid sequence of CRK-II within the vicinity of serine 41 matches with the known PAK1 phosphorylation consensus sequences [29] (Figure 4D). Notably, the RDSS amino acid sequence in CRK-II is identical to the Pak1 phosphorylation sequence in Raf. These findings establish the role of PAK1 kinase as one of the kinases that mediate CRK-II serine 41 phosphorylation.

### PAK1 kinase Suppresses *p120-catenin (CTNND1)* Promoter Activity and Expression Level

Considering the involvement of PAK1 kinase in mediating CRK-II phosphorylation on serine 41 and also the role of CRK-II serine 41 phosphorylation in *p120-catenin (CTNND1)* transcriptional repression, we asked whether PAK kinases are in fact involved in *p120-catenin (CTNND1)* repression. In order to verify this notion, we silenced PAK1 and PAK2 by siRNA and measured the *p120-catenin (CTNND1)* promoter activity in a dual luciferase assay and also measured p120-catenin protein level by western blotting (Figure 4). Following siRNA mediated silencing of PAK1 in A549 cells, we observed more than 100% increase in the luciferase activity of *p120-catenin (CTNND1)* promoter reporter compared to cells transfected with non-silencing siRNA (Figure 4A). Moreover, an increase in the p120-catenin protein level was detected in H157 and A549 cells following siRNA mediated PAK1 silencing (Figure 4C). We did not notice any significant change in the *p120-catenin (CTNND1)* promoter activity following siRNA mediated PAK2 silencing. These data further institute a role for PAK1 in transcriptional regulation of the *p120-catenin (CTNND1)*.

### Effects of PAK1 Kinase on *p120-catenin (CTNND1)* Promoter Activity is Mediated through CRK-II Serine 41 Phosphorylation

**Figure 5:**
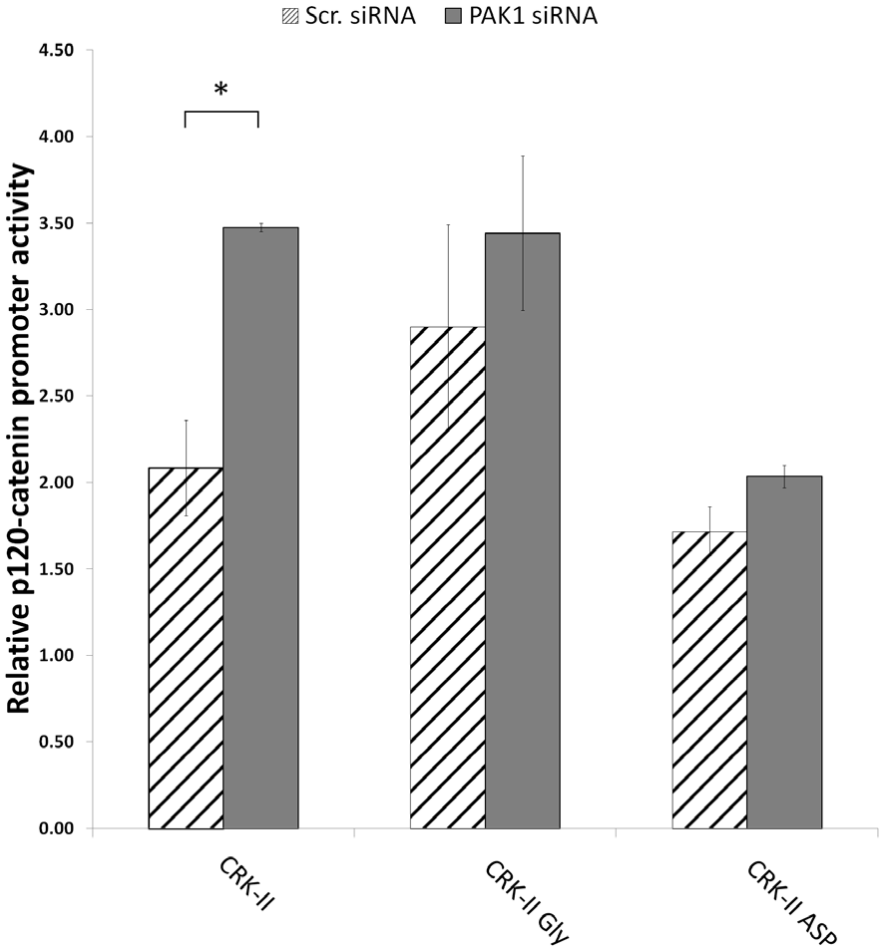
Relative *p120-catenin (CTNND1)* promoter activity in A549 cells stably expressing wild type CRK-II (WT), CRK-II (Ser41Gly) or CRK-II (Ser41Asp) mutants following siRNA mediated silencing of PAK1. Endogenous CRK is silenced in all conditions by siRNA. (2 tailed student’s t-test: * P<0.05; error bars represent ± standard deviation).

To further establish a causal relationship between PAK1 and CRK-II phosphorylation in p120-catenin expression, we decided to examine the effect of simultaneous PAK1 and CRK-II serine 41 manipulations on the *p120-catenin (CTNND1)* transcriptional regulation. Accordingly, we silenced PAK1 in A549 cells that stably express CRK-II serine 41 phosphomimetic and phosphodeficient mutants (Figure 5). We also silenced the endogenous CRK by siRNA to reduce the unwanted interactions of the endogenous CRK. As expected, A549 cells expressing wild type CRK-II showed increased level of *p120-catenin (CTNND1)* transcriptional activity following PAK1 silencing. This effect of PAK1 on *p120-catenin (CTNND1)* transcriptional activity was abrogated in cells expressing either phosphomimetic or phosphodeficient CRK-II serine 41 mutants suggesting that the inability of CRK-II to alter its phosphorylation status on serine 41 (as a result of inserted mutations) abolishes the effect of PAK1 on *p120-catenin (CTNND1)* transcription. These findings further supports the role of CRK-II serine 41 phosphorylation on PAK1 mediated *p120-catenin (CTNND1)* transcriptional regulation. The minor changes in the *p120-catenin (CTNND1)* promoter activity that are observed in CRK-II serine 41 phosphomimetic mutant expressing cells (statistically non-significant) could be as a result of the endogenous CRK-II phosphorylation alteration following PAK1 silencing. It is worth mentioning that RNAi mediated silencing of the endogenous CRK does not result is a complete knock down of the endogenous CRK-II (Figure S2).

### PAK1 Kinase Affects Cell Motility and Cell Invasiveness through CRK-II Serine 41 Phosphorylation

**Figure 6:**
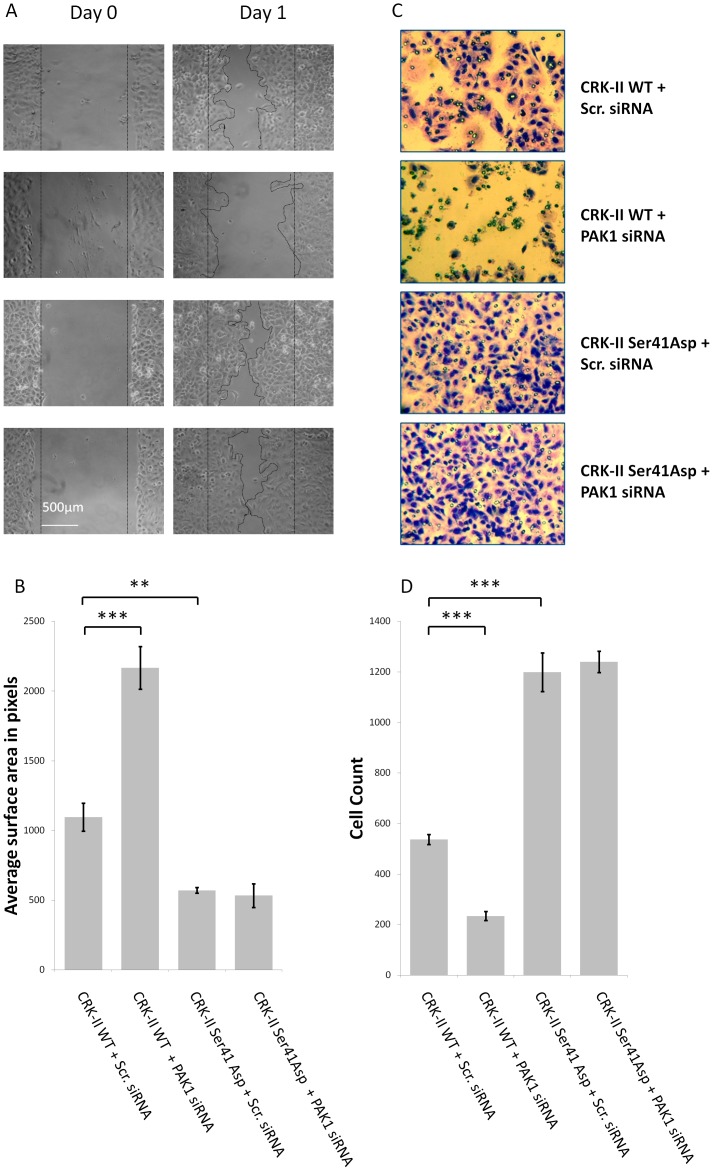
A-Wound healing assays in A549 cells stably expressing wild type CRK-II (WT) or CRK-II phosphomimetic (Ser41Asp) mutants following siRNA mediated PAK1 silencing. Endogenous CRK is silenced in all conditions by siRNA. B- Measurement of wound surface area 24 hours after the establishment of the wound among the above mentioned groups. The average is calculated following measurement of three separate experiments. (2 tailed student’s t-test: ** P<0.01; *** P<0.001; error bars represent ± standard deviation). C- Invasion assays following incubation of stably transfected A549 cells expressing wild type CRK-II (WT) or CRK-II (Ser41Asp) mutant. Each cell line was treated with PAK1 siRNA or scrambled sequence siRNA. 1×10^5^ cells were incubated over night as described in the Methods section and stained at 36 hours after incubation. D- Quantitative measurement of the invaded cells. Five separate sections of the invaded cells in each Matrigel membrane were counted. (2 tailed student’s t-test: ** P<0.01; *** P<0.001; error bars represent ± standard deviation).

Considering (i) CRK-II serine 41 phosphorylation promotes cell motility and invasiveness and (ii) PAK1 mediates CRK-II serine 41 phosphorylation, we were facing he question whether PAK1 is indeed promotes cell motility and invasiveness through CRK-II seine phosphorylation. To answer this question we examined the motility and invasiveness of A549 cells following simultaneous manipulations of PAK1 and CRK-II serine 41 phosphorylation (Figure 6). As expected, we observed a higher motility and invasiveness of A549 cells expressing CRK-II serine 41 phosphomimetic mutant compared to cells expressing wild type CRK-II. Following siRNA mediated silencing of PAK1, cells expressing wild type CRK-II showed a diminished motility and invasiveness. Interestingly, PAK1 silencing did not have any effect on the motility and invasiveness of cells expressing CRK-II serine 41 phosphomimetic mutant. Similar to other experiments, the endogenous CRK was silenced by siRNA in all conditions in order to reduce the interaction of endogenous CRK with CRK-II phosphomimetic mutant. Therefore, we conclude that PAK1 effect on promoting cell motility and invasiveness is mediated through CRK-II serine 41 phosphorylation.

## Discussion

**Figure 7:**
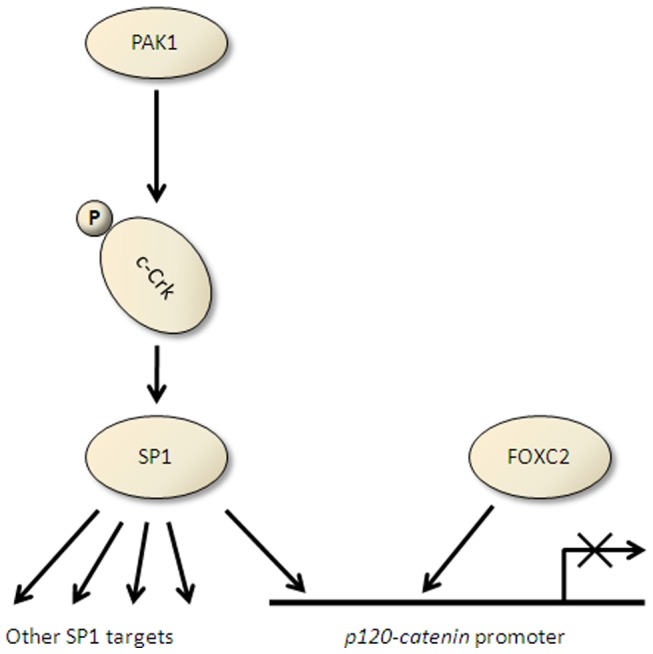
Schematic view of PAK1, CRK-II and SP1 as well as FOXC2 in transcriptional regulation of *p120-catenin (CTNND1).*

Understanding the signaling events that promote metastasis in solid tumors will help us pin point therapeutic targets in order to intervene in the metastasis process. Loss of cell-cell adhesions in epithelial cells is one of the earliest steps of tumor spread. Therefore, losing the integrity of adherens junction and its members (i.e., cadherins and catenins) plays a pivotal role in the promotion of metastasis. One of the frequent observed events in NSCLC tumors is the p120-catenin downregulation. Since p120-catenin repression in NSCLC is transcriptionally mediated [11], we decided to further investigate the upstream signaling events that eventually would lead to transcriptional repression of *p120-catenin (CTNND1)*. Through detailed analysis of the *p120-catenin (CTNND1)* promoter, the role of transcription factors FOXC2 and SP1 in the *p120-catenin (CTNND1)* downregulation were recognized. Further analysis of the binding partners of SP1 lead us identify the role of adaptor protein CRK in this pathway [12] (Figure 7). Since CRK receives input from multiple signaling pathways, it is likely that the upstream oncogenes that transmit their respective signal through CRK have a significant impact on the downregulation of p120-catenin, promotion of cell motility/invasiveness and promotion of tumor metastasis. Therefore, as a surrogate of activated upstream signals, we investigated the phosphorylation status of CRK-II and noticed CRK-II serine 41 phosphorylation has an inverse correlation with the p120-catenin expression level in a panel of NSCLC cells. Of note, this correlation was not observed with phosphorylation of CRK-II on tyrosine 221. As an adaptor protein, CRK does not contain a catalytic domain however both tyrosine and serine kinase activities have been associated with CRK [15]. Our data show CRK-II serine 41 phosphorylation manipulation alters the *p120-catenin (CTNND1)* promoter activity, expression level and also the invasive property of NSCLC cells.

Regarding CRK-I, it seems that higher expression of CRK-I oncoprotein by itself is correlated with tumorigenesis. This observation is in contrast with CRK-II where phospho-CRK-II establishes a strong association with tumorigenesis. A significant increase in CRK-I oncoprotein level and phosphorylated isoform (CRK-II) were observed in NSCLC [28]. Similarly, here we observe a close and inverse correlation between CRK-I expression as well as phospho-serine 41 CRK-II with that of p120-catenin and E-cadherin in our panel of NSCLC cells (Figure 1).

CRK serine phosphorylation is not well studied although there are anecdotal reports regarding the role of PAK family of serine/threonine kinases in CRK serine phosphorylation [20], [21]. Here we report the role of P21-activated kinase 1 (PAK1) in CRK-II serine 41 phosphorylation thereby p120-catenin downregulation and also promotion of cell motility and invasiveness in NSCLC cells. PAK family of kinases are targets of GTP binding proteins Cdc42 and Rac1 [30] and also are involved in cytoskeletal reorganization. Involvement of PAK kinases in the promotion of cell motility and cell shape are also well known [31], [32]. PAK kinases are classified in two major groups, group I (PAK1–3) and group II (PAK4–6). This classification is mainly based on the presence of an autoinhibitory region in group I PAK members [33]. The role of group I PAKs is more clearly demonstrated in cancer progression as PAK1 overexpression is seen is several tumor types. PAK1 is overexpressed in breast, ovarian, lung and head and neck cancers [25], [34]. Additionally, PAK1 expression is reportedly elevated in malignant progression of human colorectal carcinoma [35]. Interestingly, PAK1 is also engaged in wound healing and the regulation of contact inhibition in epithelial cells. Following expression of activated PAK1 in MDCK epithelial cells, a lack of growth arrest was observed upon wound closure [36]. Here, we examined the role of PAK1 and PAK2 in CRK mediated transcriptional regulation of *p120-catenin (CTNND1)* and could only identify PAK1 as a regulator of *p120-catenin (CTNND1)*. PAK1 silencing reduced CRK-II serine 41 phosphorylation in A549 and H157 cells and enhanced *p120-catenin (CTNND1)* promoter activity and protein level in NSCLC cells. Additionally, PAK1 silencing reduced cell motility and invasiveness via CRK-II serine 41 phosphorylation.

PAK1 has a number of downstream targets that regulate actin organization and polymerization, cell proliferation and apoptosis. For instance, Pak1 interacts with filamin A and LIM kinase [24], [37] which inactivate the F-actin destabilizing protein cofilin, leading to aggregation of F-actin fibers [38]. In addition to regulating the cytoskeleton, PAK1 has an important role in regulating activation of MAPK signaling pathways. Pak1 directly activates Raf-1, by phosphorylating serine 338 [39] moreover Pak1 directly activates MEK1, phosphorylating serine 298 [40]. Other than activating the ERK MAPK, overexpressed PAK1 has been reported to activate p38 MAPKs [41], [42] although the details of this interaction are not well understood. PAK1 also seems to be associated with JNK activity. Overexpression of Pak1 by different investigators has produced different reports indicating both enhanced [30], [41], [43] and impaired [44], [45] JNK activity. Furthermore, PAK1 has been reported to be a member of an anti-apoptotic signaling network via its interactions with Bad [46], [47], [48], [49]. Data provided here introduce CRK-II as another downstream target of PAK1. Considering CRK-II serine 41 is part of a PAK1 phosphorylation consensus sequence (Figure 4D), it is likely that PAK1 directly phosphorylates CRK-II on serine 41.

In summary, these data describe one of the metastatic promoting pathways in NSCLC cells involving PAK1 kinase, CRK-II, SP1 and p120-catenin. In other words, CRK-II phosphorylation seems to play a part in conveying signals downstream of PAK1. In conjunction with other report that highlights PAK1 overexpression in squamous cell NSCLC [25], [46], it seems PAK1 inhibition might provide a viable strategy in order to interrupt the pro-metastatic behavior of certain NSCLC subtypes.

## Materials and Methods

### Cell Cultures

A549, H157, Rh2 and H358 cells were routinely cultured in RPMI supplemented with antibiotics and 10% heat-inactivated FBS (Omega Scientific, Tarzana, CA). Immortalized normal human epithelial cells (BEAS-2B) were cultured in BEBM medium supplemented with all the additives (Lonza/Clonetics Corporation, Switzerland; Catalog No. CC-3170).

### Measurement of p120ctn Promoter Activity by Dual Luciferase Assay

*p120ctn* promoter construct was transfected into NSCLC cell lines by Lipofectamine 2000 (Invitrogen). Twenty-four hours after transfection cells were washed with PBS and lysed using a Branson Sonifier in 1x passive lysis buffer (Promega) at room temperature (RT). Reporter gene expression was assessed by using the Dual-Luciferase Reporter Assay System kit (Promega) according to manufacturer’s instructions in a TD-20/20 Luminometer (Turner Biosystems, Sunnyvale, CA). We normalized for transient transfection efficiency (i.e. firefly luciferase activity) by co-transfection of a *Renilla* luciferase expressing control vector (pRL-SV40). All experiments were performed in triplicate and were reported as means ± standard deviation (SD), and each experiment was performed at least twice.

### siRNA Mediated Gene Silencing

A549 and H157 cells were transfected with siRNA against CRK, PAK1 and PAK2. Transfections were performed by using Lipofectamine 2000 (Invitrogen). The siRNA duplex sequences that we used to silencing the above mentioned genes are as follows:

- CRK: 5′-CUGCUUACCCUGAUUUAUUdtdt-3′
  5′-AAUAAAUCAGGGUAAGCAUdtdt-3′.
- PAK1: 5′-GAAAGAGCGGCCAGAGAUUdtdt-3′
  5′-AAUCUCUGGCCGCUCUUUCdtdt-3′.
- PAK2: 5′-GGAUUUCUUAAAUCGAUGUdtdt-3′
  5′-ACAUCGAUUUAAGAAAUCCdtdt-3′.

As a control, scrambled non-silencing siRNA was used (Sigma-Aldrich Cat. number SIC002-10NMOL). Concentrations of siRNA were kept at 100 nM among groups. In case of silencing the endogenous CRK, the above siRNA is directed against CRK mRNA in a region outside the open reading frame (approximate siRNA start site 1210 based on reference NM_005206). Therefore using this CRK siRNA has no effect on the CRK expressing plasmids.

### Measurement of mRNA Level by qRT-PCR

We used TaqMan probes and SYBR Green methods for measurement of mRNA level following gene silencing. In case of p120ctn, mRNA level in NSCLC cell lines were measured by TaqMan probe; assay IDs: Hs00931670_m1 (Applied Biosystems, Foster City, CA). For CRK, PAK1 and PAK2 we used SYBR Green method with the following primers sets.

- PAK1-forward: 5′-CGTGGCTACATCTCCCATTT-3′
- PAK1-reverse: 5′-TCCCTCATGACCAGGATCTC-3′
- PAK2-forward: 5′-GAAGAATCCTCAGGCTGTGC-3′
- PAK2-reverse: 5′-GGGTCACCTATGCTCACGAT-3′
- CRK-forward: 5′-GCAAGAGAGGGATGATTCCA-3′
- CRK-reverse: 5′-ATGGGAAGTGACCTCGTTTG-3′
- Actin-forward: 5′-TGACGGGGTCACCCACACTGTGCCCATCTA-3′
- Actin-reverse: 5′-CTAGAAGCATTTGCGGTGGACGATGGA-3′

In each cell type, total RNA was extracted by using Trizol method and cDNA was prepared by an RT-PCR reaction using SuperScript-II RT Kit (Invitrogen) and Oligo (dT)s according to the manufacturer’s instructions. For each cDNA sample an internal control (beta actin) was also measured by TaqMan probe. In order to compare the expression level of mRNA between samples, we used the Comparative Ct Method (ΔΔCt). Relative expression of mRNA compared to beta actin in each sample was calculated (ΔCt) and relative expression of mRNA among samples was determined by calculating the difference in (ΔCt) between samples (ΔΔCt). All relative quantitative PCRs were performed, recorded, and analyzed by using the ABI 7300Prism Sequence Detection System (Applied Biosystems, Foster City, CA). All samples were carried out in triplicate (10 ng of total RNA per well) and repeated at least twice. Controls without template or reverse transcriptase were run in each experiment.

### Western Blots

NSCLC cell lines were seeded in 10 cm Petri dishes at 5×10^5^ cells per dish, which resulted in 30–40% confluency 24 hours after plating. Cells were harvested at 24 hours by adding trypsin, pelleted and lysed in 100 µl of lysis buffer (NaCl 15 mM; EDTA 0.5 mM; Tris 10 mM) using a Branson Sonifier. Cell debris was collected by centrifugation at 4°C, and protein concentration was measured by the BCA method. Protein was resolved by SDS-PAGE and was transferred to a nitrocellulose membrane. The membrane was blocked with TBS with 5% nonfat powdered milk.

Membranes were immunoblotted with the following primary antibodies: p120ctn (BD biosciences Cat. number 610133); E-cadherin (BD biosciences Cat. number 61081); CRK II (Santa Cruz Biotechnology Cat. number sc-289); p-Ser41 CRK-II (Santa Cruz Biotechnology Cat. number sc-130186); PAK1 (Sigma-Aldrich Cat. number SAB4300427) and c-Myc (Santa Cruz Biotechnology Cat. number sc-40).

Horse radish peroxidase conjugated secondary antibodies were used for detection of bands by chemiluminescence (ECL western blotting detection reagents, Amersham Biosciences, Piscataway, NJ, USA).

### CRK Mutant Construct Generation and Stable Expression

We obtained a CRK-II expression plasmid (OriGene Technologies Cat. number RC201701). A sight directed mutagenesis kit (Strategene Cat. number200523) was used according to the manufacturer instructions in order to replace serine 41 with either glycine or aspartic acid by replacing the corresponding nucleotides. The following primer sets were used for site directed mutagenesis.

- Ser41Gly Forward: 5′-TCC TGG TGC GGG ACT CGG GCA CCA GCC CCG GGG ACT ATG TGC-3′
- Ser41Gly Reverse: 5′-GCA CAT AGT CCC CGG GGC TGG TGC CCG AGT CCC GCA CCA GGA-3′
- Ser41Asp Forward: 5′-TCC TGG TGC GGG ACT CGG ACA CCA GCC CCG GGG ACT ATG TGC-3′
- Ser41Asp Reverse: 5′-GCA CAT AGT CCC CGG GGC TGG TGT CCG AGT CCC GCA CCA GGA-3′

All constructs were checked by sequencing for accuracy of mutagenesis. Subsequently, A549 cells were transfected with the above mentioned plasmids by using Lipofectamine 2000 (Invitrogen). 24 hours after transfection, cells were treated with G418 at 1500 µg/ml in order to generate stable cell lines. The pooled transfected and selected cells were used for the subsequent experiments.

### Wound Healing Assays and Microscopy

A549 and H157 cells were plated in a 6 well plate dish at 1×10^5^ cells per well and were grown to confluent stage. By using a sterile P1000 pipette tip, a straight scratch was made along the largest diameter of each well and a baseline photomicrograph was taken from this scratch with two different magnifications. A follow up photomicrograph was taken at 24 hours. Photomicrographs of the cells were obtained by a Nikon Eclipse TS100 inverted microscope equipped with a Cannon A510 digital camera. The digital camera was connected with a Max View Plus adaptor (ScopeTronix) to the inverted microscope. An equal area of the photomicrographs from each condition was imported into the Adobe Photoshop software and the resolution of the pictures were set at 25 pixels/inch. The resulting low-resolution pictures were made of countable squares pixels. The surface area of the wound in each condition was measured by counting the number of pixels in each wound area. Experiments were repeated three times and the average wound surface area was compared among the groups.

### Cell Invasion Assay

Matrigel cell invasion assays were performed by using Matrigel Invasion Chamber (BD Biosciences Cat. number354480) according to the manufacturer instructions. 1×10^5^ cells were incubated in the upper chambers in RPMI in the absence of serum in triplicates. As the chemoattractant, RPMI with 10% FBS was used in the lower chambers. Cells were incubated for 24–36 hours in the 37°C incubator. Cells were harvested and stained with Diff-Quik™ kit according to the manufacturer’s instructions. Photomicrographs were taken by an Olympus BH2 microscope equipped with a Cannon A510 digital camera. The invaded cells in five corresponding regions of each Matrigel membrane were counted and the mean of invaded cells were compared among the groups.

### Measurement of Cell Proliferation

Following plating equal number of A549 cells transfected with CRK-II and CRK-II mutants in triplicates, cells were harvested at 24 hour intervals. Cells were stained by Trypan blue and counted by using a hemocytometer.

### Measurement of Western Blot Signal Intensity

A photograph of the western blots were imported into Adobe Photoshop software. The photographs were inverted in order to present the protein bands as the brighter signal compared to the background. An equal area of each band was selected and the mean and standard deviation of signal intensity were recorded by the histogram tool of the software. In case the signal intensity of any selected band was reaching the maximum range of the histogram thereby causing signal saturation, the contrast of the image was adjusted to avoid signal saturation.

## Supporting Information

Figure S1 **Western blots showing the endogenous CRK-II and CRK-II-Myc mutants following transient transfection of CRK-II-Myc mutant constructs in A549, Rh2, and H157 cells.** Measurement at 48 hours post transfection.
(TIF)

Figure S2 **Western blots showing the endogenous CRK-II and CRK-II-Myc mutants in A549 cells stably transfected with CRK-II mutants.** The endogenous CRK-II is diminished by a CRK-II siRNA directed against a sequence outside the open reading frame of *CRK*. The expression level of CRK-II-Myc mutants as well as the endogenous CRK-II levels are quantified and the ratio of CRK-II mutant/endogenous CRK-II are demonstrated.
(TIF)

Figure S3 **Wider views of wound healing assays presented in (** **Figure 3** **).**
(TIF)

Figure S4 **Wider views of the invasion assays presented in (** **Figure 3** **).**
(TIF)

Figure S5 **Line chart representing the growth rate of A549 cells stably expressing pCMV (empty vector), wild type CRK-II (WT), CRK-II (Ser41Gly) or CRK-II (Ser41Asp) mutants.**
(TIF)
