# Supplementary figures and images for: PAK1 Kinase Promotes Cell Motility and Invasiveness through CRK-II Serine Phosphorylation in Non-Small Cell Lung Cancer Cells
Source: PLoS One. 2012 Jul 27;7(7):e42012. doi: 10.1371/journal.pone.0042012 (PMC3407072; doi:10.1371/journal.pone.0042012)

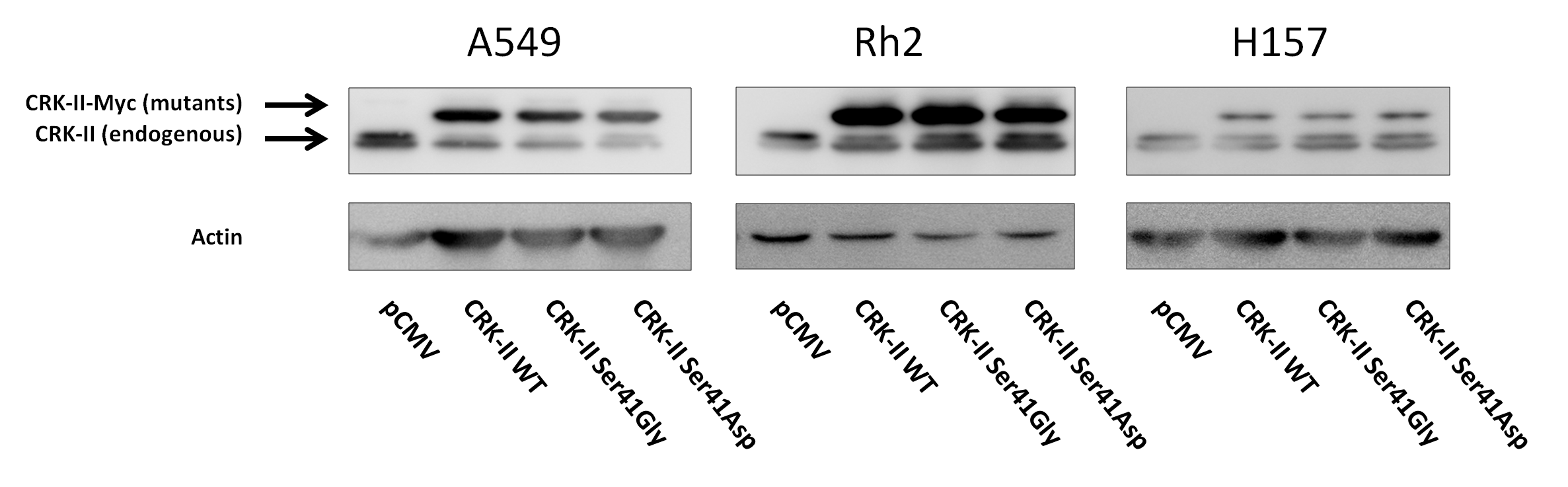

Supplement: Figure S1 — Western blots showing the endogenous CRK-II and CRK-II-Myc mutants following transient transfection of CRK-II-Myc mutant constructs in A549, Rh2, and H157 cells. Measurement at 48 hours post transfection. (TIF) [file pone.0042012.s001.tif]

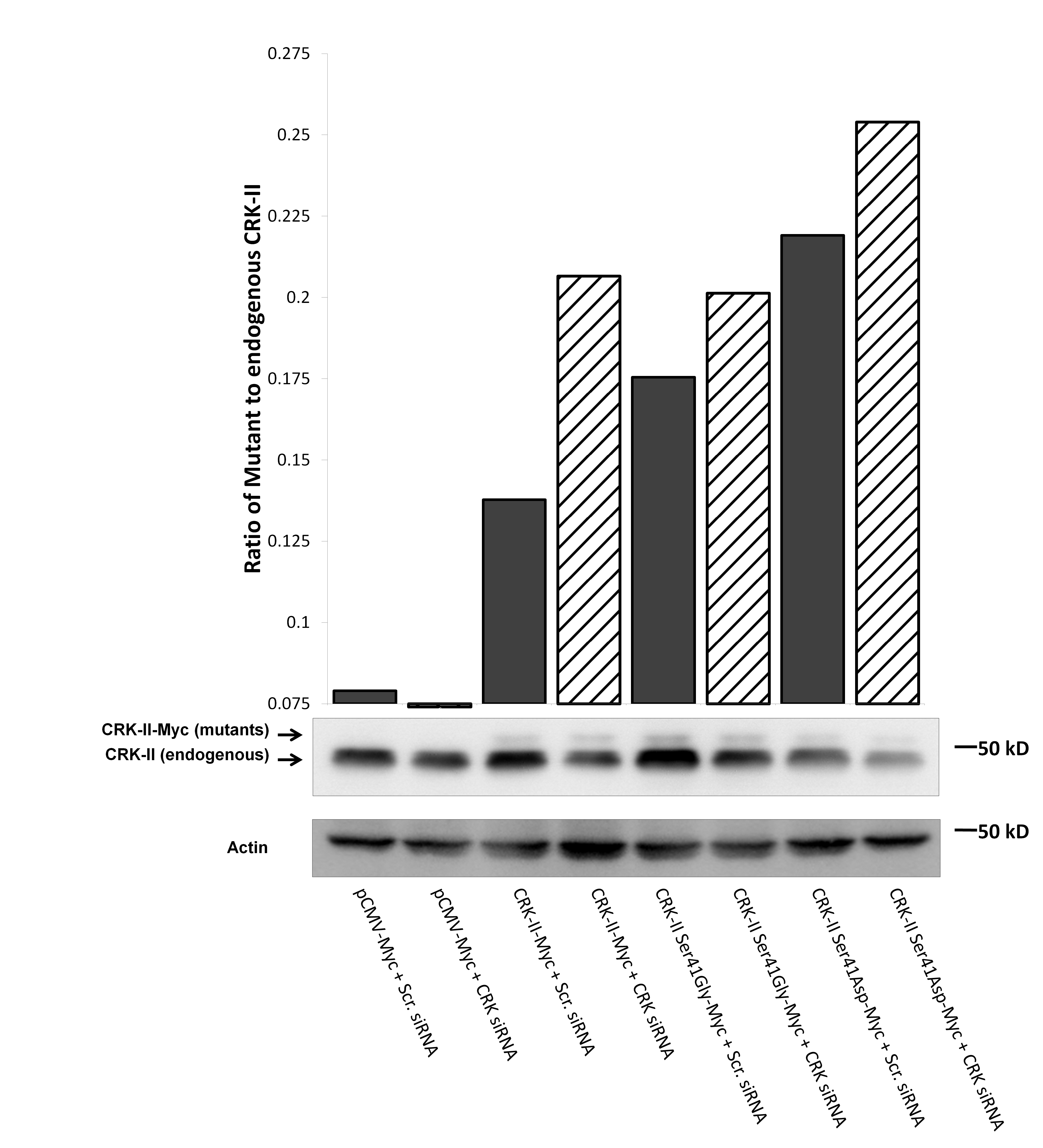

Supplement: Figure S2 — Western blots showing the endogenous CRK-II and CRK-II-Myc mutants in A549 cells stably transfected with CRK-II mutants. The endogenous CRK-II is diminished by a CRK-II siRNA directed against a sequence outside the open reading frame of CRK. The expression level of CRK-II-Myc mutants as well as the endogenous CRK-II levels are quantified and the ratio of CRK-II mutant/endogenous CRK-II are demonstrated. (TIF) [file pone.0042012.s002.tif]

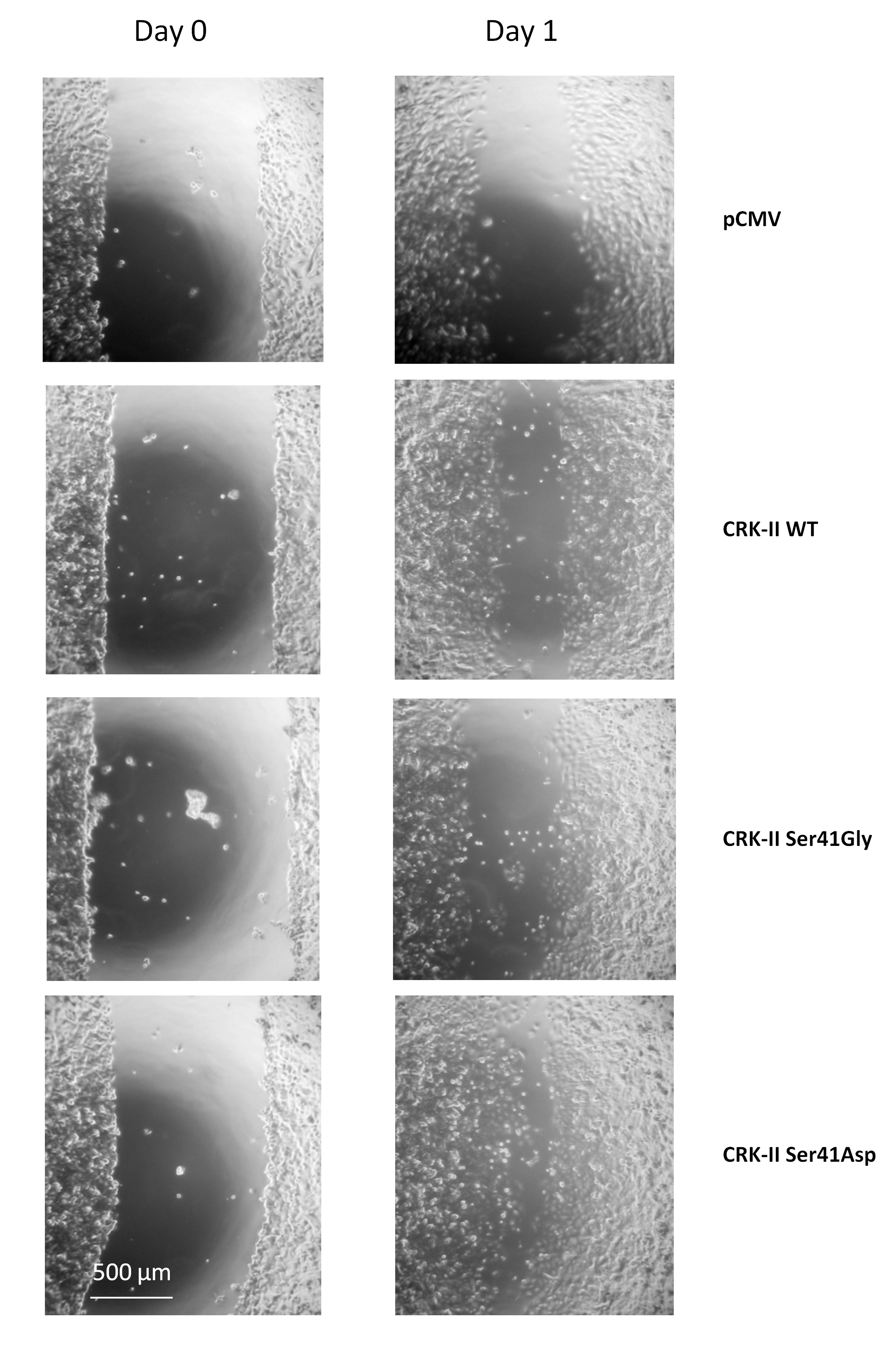

Supplement: Figure S3 — Wider views of wound healing assays presented in ( Figure 3 ). (TIF) [file pone.0042012.s003.tif]

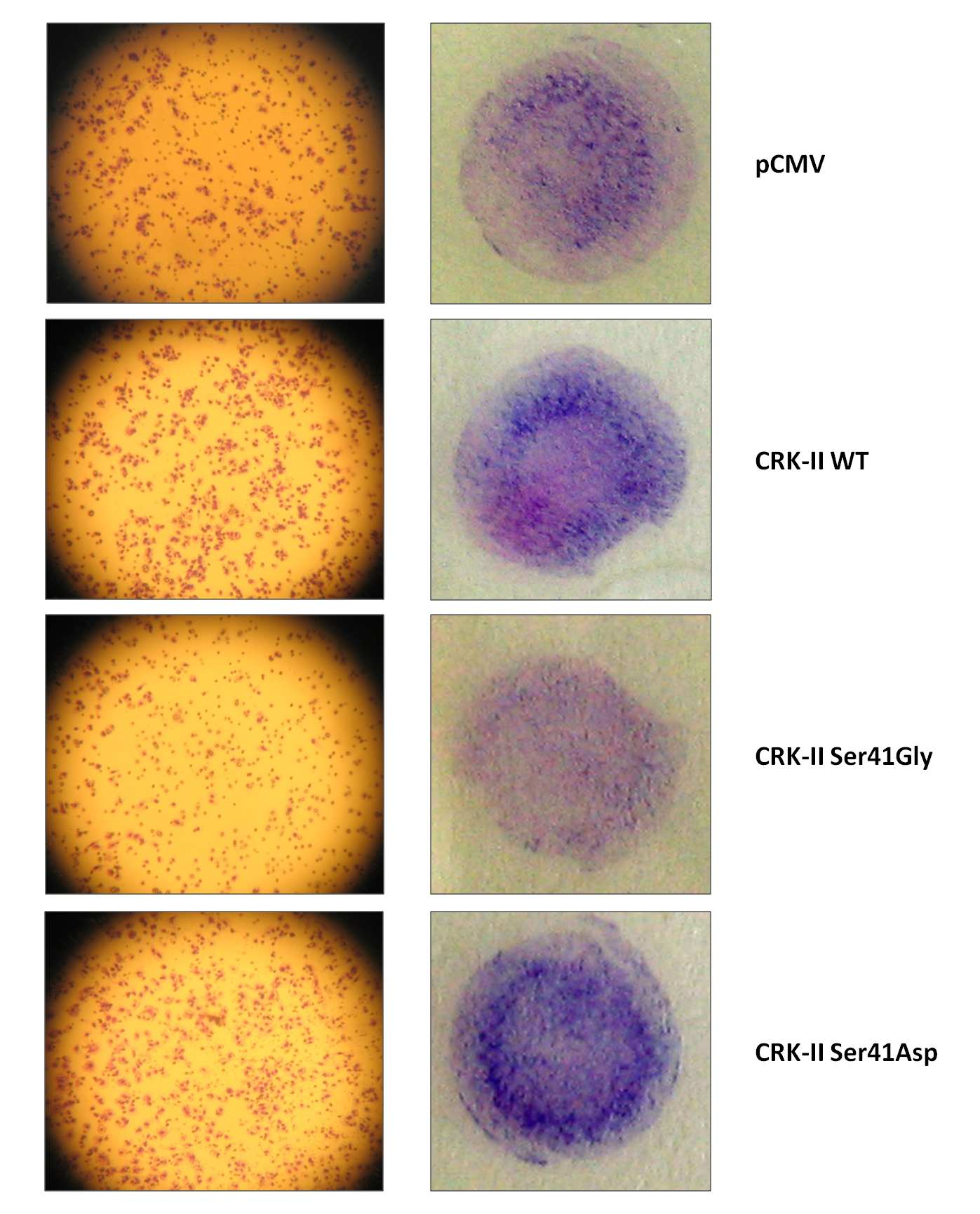

Supplement: Figure S4 — Wider views of the invasion assays presented in ( Figure 3 ). (TIF) [file pone.0042012.s004.tif]

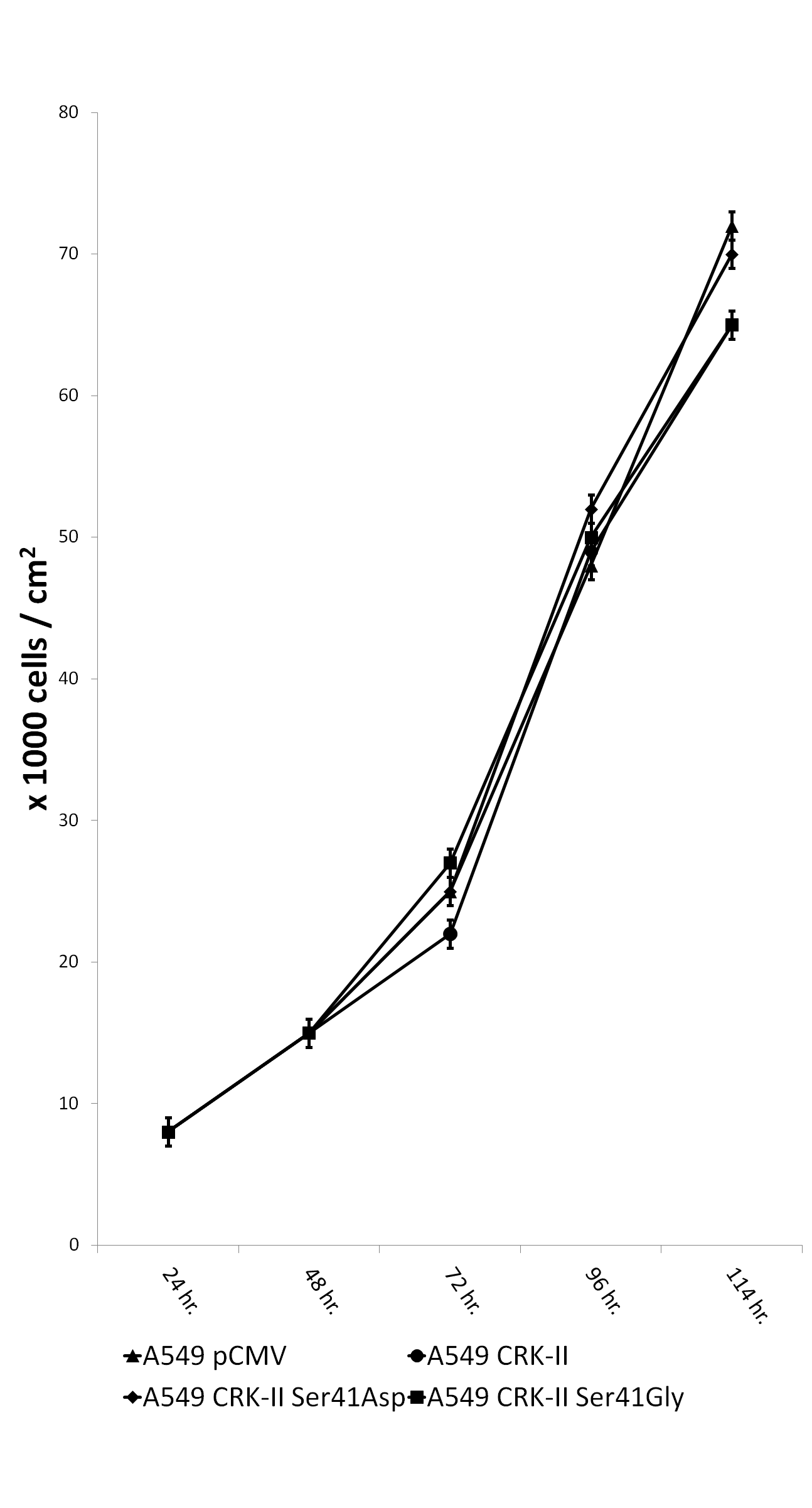

Supplement: Figure S5 — Line chart representing the growth rate of A549 cells stably expressing pCMV (empty vector), wild type CRK-II (WT), CRK-II (Ser41Gly) or CRK-II (Ser41Asp) mutants. (TIF) [file pone.0042012.s005.tif]
